# Delayed vs. immediate placement of restorative materials over Biodentine and RetroMTA: a micro-shear bond strength study

**DOI:** 10.1186/s12903-024-03917-3

**Published:** 2024-01-25

**Authors:** Ozge Celiksoz, Ozgur Irmak

**Affiliations:** 1 Department of Restorative Dentistry, Faculty of Dentistry, Eskisehir Osmangazi University, Eskisehir, Turkey; 2 Department of Restorative Dentistry, Faculty of Dentistry, Cyprus Health and Social Sciences University, Guzelyurt, Cyprus

**Keywords:** Dental pulp capping, Dental cavity liner, Permanent dental restorations, Pulpotomy

## Abstract

**Background:**

The aim of the present study was to investigate the micro-shear bond strength (µSBS) of various restorative materials applied on two different fast-setting calcium silicate-based materials and to evaluate the effect of restoration time on µSBS.

**Methods:**

A total of 180 acrylic blocks with a cavity in the center were randomly divided into 2 main groups according to the capping material to be used (Biodentine or RetroMTA). The specimens were also randomly divided into 3 groups according to the restoration time (3 min, 12 min, 24 h). After the specified waiting periods, glass hybrid material (EQUIA Forte HT), resin composite (Filtek Z550) and light-cured calcium silicate material (Theracal LC) were placed on the specimens with tygon tubes. The specimens were kept for 24 h and then subjected to µSBS test. Statistical analysis was performed by 3-way ANOVA followed by Tukey test for pairwise comparisons (α = 0.05).

**Results:**

There is a statistically significant difference (*p* < 0.05) between the µSBS values obtained by applying resin composite on RetroMTA after different setting times (24 h > 12 min > 3 min). The µSBS obtained for the Biodentine-resin composite at the end of the 3 min setting time is significantly lower (*p* < 0.05) than the µSBS values at 12 min and 24 h. For both calcium silicate cements, at the end of all time periods, the µSBS obtained when resin composite was applied at the end was higher than the other materials (*p* < 0.05).

**Conclusions:**

For Biodentine-resin composite bonding, the manufacturer’s recommendation of 12 min can be considered an appropriate time, whereas for RetroMTA-resin composite bonding, the µSBS increased as the waiting time increased. Regardless of the waiting time, it is recommended to prefer direct composite resin restoration over Biodentine and RetroMTA.

## Introduction

In an attempt to preserve vitality of inflamed dental pulp, more conservative treatments have been advocated instead of root canal treatment [1, 2]. Vital pulp treatments (VPTs) aim to preserve pulpal viability by covering the exposed pulp with a biocompatible and bioactive material to reduce and halt pulpal inflammation and prevent further bacterial leakage [3]. In the past, the material most frequently utilized for VPT was calcium hydroxide (Ca(OH)_2_) [4]. However, studies found that Ca(OH)_2_ does not adhere to to dentin, has poor sealing ability, degrades over time, and may result in the creation of defective dentin bridge [5, 6]. To overcome these disadvantages related to calcium hydroxide, calcium silicate-based biomaterials have been developed for use in VPTs. Mineral trioxide aggregate (MTA), which is predecessor one of these calcium silicate-based cements (CSCs); demonstrates a variety of clinical and biological benefits such as improved sealing, biocompatibility, antibacterial activity and stimulation of bioactive endogenous molecules. Due to these benefits, it has become the new ‘gold standard’ against which new materials are compared, replacing calcium hydroxide in VPT [7]. However, traditional MTA has disadvantages such as difficulty in application, potential for dental tissue discoloration and long setting time [8]. A second dental visit could be necessary to finish the final restoration due to the long setting time of the material. This delayed restorative treatment raises costs, prolongs the chair time, and increases the risk of failure of VPT [3, 8]. In addition, a second appointment is not desirable for uncooperative patients such as children and the disabled [9]. New generation of calcium silicate-based biomaterials have been introduced, which set in a shorter time and have less discoloration potential compared to traditional MTA [8, 10–12]. These materials are called fast-setting calcium silicate-based cements (FSCSCs). One of these relatively new biomaterials is Biodentine (Septodont, France), introduced in 2011. The advantages of Biodentine include increased sealing ability, high compressive strength, better biomineralization capacity and biocompatibility. Its discoloration potential is less than MTA [6, 13]. Its capsule form allows for a more standardized mixing and easier application [13]. Biodentine has initial setting time of approximately 9–12 min thus makes it suitable for completion of the final definitive restoration in a single session [14]. Another fast setting material is RetroMTA (BioMTA, Republic of Korea). The content of calcium carbonate in RetroMTA and the formation of calcium zirconia complexes after mixing reduces setting chemistry and setting time and improves its mechanical properties [15]. Its manufacturer states the initial setting time as 3 min and final setting time as 12 min [16]. This time makes a big difference between other CSCs. Researchers have mainly focused on the pulpal consequences of these materials [17]. However, the placement of a permanent and a well-sealed restoration has a direct impact on the outcome of VPT. Therefore, in addition to the bond strength between the restorative material and the tooth hard tissues, the bond strength between the VPT material and the restorative material is also important [13, 17]. In the literature, there are limited number of studies evaluating the placement time of the definitive restoration over these FSCSCs [18, 19]. Thus the aim of this study was to evaluate the effect of permanent restoration placement time on the bond strength between different restorative materials placed over different FSCSCs. The first null hypothesis tested was that the restorative material placement time would not have effect on bond strength between the restorative material and FSCSC. Second null hypothesis tested was the type of the restorative material would not have effect on bond strength between the restorative material and FSCSC.

## Materials and methods

**Fig. 1:**
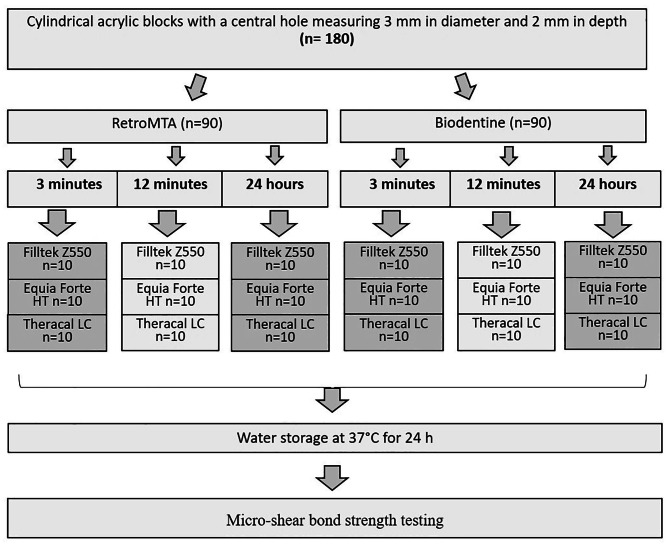
Study workflow

All stages of the experiments were performed by a single operator. The materials and their contents are given in Table 1. The workflow of the study is presented in Fig. 1. The power analysis was performed with PASS software (PASS 11. NCSS, LLC, USA) and the sample size was calculated as *n* = 10. (Type I error level = 0.05) The power of the study was determined as 92.58%.

**Table 1:** Compositions and manufacturer’s instructions of materials investigated in the present study

Materials	Manufacturer	Components	Instructions for use
RetroMTA **Lot number**: RM1604D15	BioMTA, Seoul, Republic of Korea	Powder: Calcium carbonate, Silicon oxide, Hydraulic calcium zirconia complex, Aluminum oxide Liquid: Distilled water	Pour the 0.3 g powder onto the three drops of distilled water and wet it gently for 20 s. Wait until the shiny surface disappears, then apply it with a carrier.
Biodentine **Lot number**: B25005	Septodont; St Maure des Fosses, France	Powder: Tri-calcium silicate, Di-calcium silicate, Calcium carbonate and oxide, Iron oxide, zirconium oxide Liquid: Calcium chloride, Hydro soluble polymer	One dose of liquid and powder mixed for 30 s at 4000 rpm in an amalgamator
Clearfil SE Bond **Lot number**: 101,752	Kuraray Noritake Dental Inc., Tokyo, Japan	Primer 10-MDP, HEMA, hydrophilic dimethacrylate, photoinitiator, water, hydrophilic dimethacrylate Bond 10-MDP, Bis-GMA, HEMA, hydrophilic dimethacrylate, photoinitiators, aromatic tertiary amine and silanated colloidal silica	1. Apply primer for 20s 2. Dry with mild air flow 3. Apply bond and mild air for 5s 4. Light cure for 10s
Filtek Z550 **Lot number**: N728631	3 M ESPE; St Paul, MN, USA	Bis-GMA, UDMA, Bis-EMA, TEGMA, and PEGDMA, surface-modified zirconia/silica fillers 3000 nm (3 μm or less), nonagglomerated/nonaggregated surface-modified silica particles 20 nm	Place and light cure restorative in increments.
EQUIA Forte HT **Lot number**: 2,012,181	GC; Tokyo, Japan	Fluoroaluminosilicate glass, polyacrylic acid, iron oxide polybasic carboxylic acid, water	Shake the capsule, push the plunger until it is fully depressed with the main body and hold it down for 2 s. Immediately set it into a mixer and mix for 10 s (+/- 4,000 RPM). Immediately remove the mixed capsule from the mixer and load it into the GC capsule applier. Make two clicks to prime the capsule then syringe.
Theracal LC **Lot number**: 1,900,006,871	Bisco; Schaumburg, IL, USA	CaO, calcium silicate particles, Sr glass, fumed silica, barium sulphate, barium zirconate, bis-GMA, polyethylene glycol dimethacrylate	Apply the Theracal LC in incremental layers. Layer is not to exceed 1 mm in depth. Light cure between layers. Light cure each increment for 20 s.

### Specimen preparation

A self-cure acrylic material was mixed and placed into a hollow cylindrical block measuring 11 mm in diameter and 35 mm in height. While the acrylic material was being cured, silicone discs with a diameter of 3 mm and a depth of 2 mm were embedded centrally into the acrylic provided that top of the blocks were flush with the topmost surface of the cylindrical block. After the self-cure acrylic was completely cured, embedded silicone discs were removed and standardized cavities were obtained. A total of 180 standardized cavities were prepared. Cavities were then randomly and equally divided into two main groups according the FSCSC to be used. Ninety of the cavities were completely filled with RetroMTA and other ninety were completely filled with Biodentine. All FSCSCs were mixed according to the manufacturer’s instructions and applied with appropriate hand instruments. A glass slab was placed over the samples to create a standard surface. Both RetroMTA- and Biodentine-filled cavities were randomly and equally divided into 3 subgroups according to the restoration placement time (3 min, 12 min, 24 h). Each subgroup was further divided into 3 subgroups according to the type of restorative material to be applied (*n* = 10).

### Restorative material placement over FSCSCs

FSCSC-filled cavities were covered with moist sponge and kept in an 100% humid environment at 37 °C until the restoration placement. Three different restorative materials, one nano-hybrid resin composite material, one glass hybrid restorative material, one light-cured calcium silicate cement were placed over the FSCSCs. During the restoration, the sponge on the specimens was removed and no surface treatment was performed on the cement surface. According to the experimental subgroups, restorative materials were placed either 3 min, 12 min or 24 h after placement of FSCSC.

#### Application of the nano-hybrid composite material

A two step self-etch adhesive (Clearfil SE Bond, Kuraray, Japan) was applied over the FSCSC according to the manufacturer’s recommendations, and light cured using LED light curing unit (BluePhase, Ivoclar Vivadent, Lichtenstein) with radiant emittance of 1200 mW/cm^2^. A flexible polymer tubing (Tygon tube) with 1.6 mm inner diameter, 5 mm outer diameter and 2 mm height (Interlab AS, Turkey) were placed on the adhesive applied surface, then the nano-hybrid composite resin (Filtek Z550, 3 M ESPE, USA) was filled into the tubes with a hand instrument and cured according to the manufacturer’s recommendations with the same LED light curing unit.

#### Application of the glass hybrid restorative material

Glass hybrid restorative material (EQUIA Forte HT, GC Corporation, Japan) was mixed according to the manufacturer’s instructions and applied into the tube placed on the surface of the FSCSC.

#### Application of the light-cured calcium silicate cement

Light-cured calcium silicate cement (Theracal LC, Bisco, USA) was applied in 2 layers of 1 mm increments into the tube placed on the surface of the FSCSC in accordance with the manufacturer’s instructions. Each layer was polymerized with same LED light curing unit.

All specimens were then stored in distilled water at 37 °C for 24 h. At the end of the storage period, tubes were carefully removed with a sharp scalpel and discarded.

### Micro-shear bond strength testing

A universal Testing Machine (MOD Dental MIC-101, Esetron Smart Robotechnologies, Turkey) using a metal jig with a crosshead speed of 0.5 mm/min and a 50 kg load cell were used for micro-shear bond strength **(**µSBS) testing. Fracture loads were recorded in Newtons (N) and converted to Megapascals (MPa) by dividing the recorded load to the surface area of the debonded restorative material. Debonded surface of each specimen was examined under a stereomicroscope (Model M80; Leica Microsystems Ltd., Switzerland) at 30x magnification. Defects were classified as adhesive (failure at the interface), cohesive (failure within the FSCSC or restorative material), mixed (a combination of adhesive and cohesive failures). Select samples of each fracture type were examined with scanning electron microscopy (JEOL JSM-5600LV) at 20 kV, 33x and 50x magnification after gold plating.

### Statistical analysis

Three-way analysis of variance (ANOVA) and the Tukey post hoc test used. A statistical analysis software (Jamovi v1.6, The Jamovi Project, https://www.jamovi.org) was used for the analyses (α = 0.05).

## Results

**Fig. 2:**
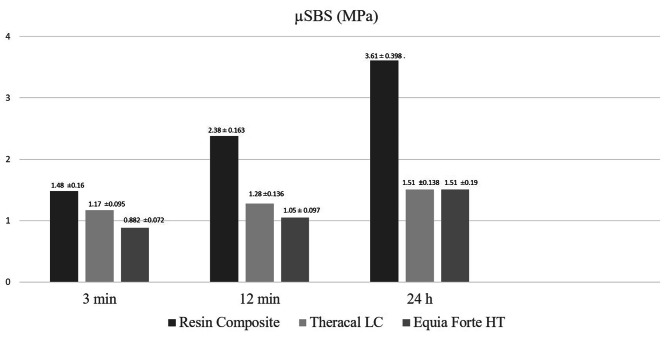
The bond strength of the restorative materials with RetroMTA according to time periods

**Fig. 3:**
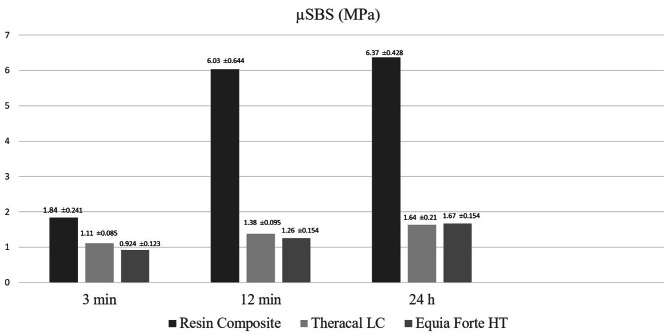
The bond strength of the restorative materials with Biodentine according to time periods

Table 2 shows, for each FSCSC, the mean (± SD) µSBS values of the restorative materials placed over FSCSCs at different times. Table 3 shows, for each restorative material, the mean (± SD) µSBS obtained between the FSCSC and the restorative material at the end of each setting time. The bond strength of the restorative materials with RetroMTA according to time periods is shown in Fig. 2 and the bond strength of the restorative materials with Biodentine according to time periods is shown in Fig. 3. Accordingly, the µSBS values obtained when resin composite (RC) was applied over RetroMTA increased as the waiting time increased (24 h > 12 min > 3 min) (*p* < 0.05). The µSBS values obtained by applying Theracal LC over RetroMTA were statistically similar regardless of the time (*p* > 0.05). When EQUIA Forte HT was applied over RetroMTA, the highest µSBS values were obtained after 24 h (*p* < 0.05).

**Table 2:** For each FSCSC, the mean (± SD) µSBS values of the restorative materials placed over FSCSCs at different times

FSCSC	Restorative Material	Time		
		**3 min (MPa** ± **SD)**	**12 min (MPa** ± **SD)**	**24 h (MPa** ± **SD)**
RetroMTA	Resin Composite	1.48 ± 0.16 ^Aa^	2.38 ± 0.163 ^Ba^	3.61 ± 0.398 ^Ca^
	Theracal LC	1.17 ± 0.095 ^Aab^	1.28 ± 0.136 ^Ab^	1.51 ± 0.138 ^Ab^
	EQUIA Forte HT	0.882 ± 0.072^Ab^	1.05 ± 0.097 ^Ab^	1.51 ± 0.19^Bb^
Biodentine	Resin Composite	1.84 ± 0.241^Aa^	6.03 ± 0.644 ^Ba^	6.37 ± 0.428 ^Ba^
	Theracal LC	1.11 ± 0.0852 ^Ab^	1.38 ± 0.0954 ^ABb^	1.64 ± 0.21 ^Bb^
	EQUIA Forte HT	0.924 ± 0.123 ^Ab^	1.26 ± 0.154 ^Ab^	1.67 ± 0.154 ^Bb^

Means sharing a superscript letter are not significantly different (*p* > 0.05)

Uppercase letters compare means in each row

Lowercase letters compare means in each column

**Table 3:** For each restorative material, the mean (± SD) µSBS obtained at the end of each setting time between the FSCSC and the restorative material

Restorative Material	FSCSS	Time		
		3 min (MPa ± SD)	12 min (MPa ± SD)	24 h (MPa ± SD)
Resin Composite	RetroMTA	1.48 ± 0.16 ^A^	2.38 ± 0.163 ^A^	3.61 ± 0.398 ^A^
	Biodentine	1.84 ± 0.241^A^	6.03 ± 0.644 ^B^	6.37 ± 0.428 ^B^
Theracal LC	RetroMTA	1.17 ± 0.095 ^A^	1.28 ± 0.136 ^A^	1.51 ± 0.138 ^A^
	Biodentine	1.11 ± 0.0852 ^A^	1.38 ± 0.0954 ^A^	1.64 ± 0.21 ^A^
EQUIA Forte HT	RetroMTA	0.882 ± 0.072 ^A^	1.05 ± 0.097 ^A^	1.51 ± 0.19 ^A^
	Biodentine	0.924 ± 0.123 ^A^	1.26 ± 0.154 ^A^	1.67 ± 0.154 ^A^

Means sharing a superscript letter are not significantly different (*p* > 0.05)

Per restorative material, letters compare column wise the mean µSBS obtained between the FSCSCs and each restorative material separately per setting time

When RC was applied over Biodentine, the lowest µSBS values were obtained at the end of the 3 min waiting time, and there was no significant difference between the µSBS values at the 12 min and 24 h waiting times (*p* > 0.05). There was no difference between the µSBS values obtained after 3 and 12 min by applying Theracal LC over Biodentine (*p* > 0.05). When EQUIA Forte HT was applied over Biodentine, the highest µSBS values were observed after 24 h waiting time (*p* < 0.05).

Amongst the restorative materials placed over any FSCSC, at the end of all time periods, µSBS obtained with RC was highest compared to those of the other restorative materials (*p* < 0.05).

After 12 min or 24 h, RC over Biodentine had significantly higher µSBS values than µSBS of RC over RetroMTA (*p* < 0.05). Theracal LC or EQUIA Forte HT applied over both FSCSCs, showed similar µSBS values regardless of time (*p* > 0.05).

**Fig. 4:**
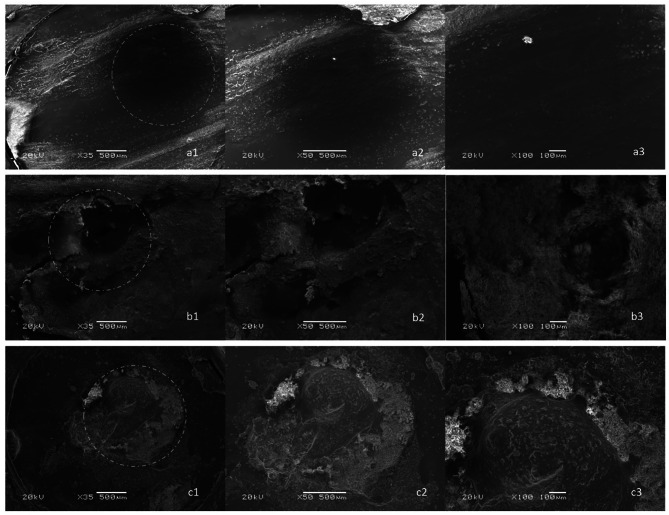
SEM images of failure types for RetroMTA material. The fracture zone is marked with a circle. Images indicated with the letter ‘a’ represent adhesive fracture. a1 indicates x35 magnification, a2 indicates x50 magnification, a3 indicates x100 magnification. Images indicated with the letter ‘b’ represent cohesive fracture. b1 indicates x35 magnification, b2 indicates x50 magnification, b3 indicates x100 magnification. Images indicated with the letter ‘c’ represent mix fracture. c1 indicates x35 magnification, c2 indicates x50 magnification, c3 indicates x100 magnification

**Fig. 5:**
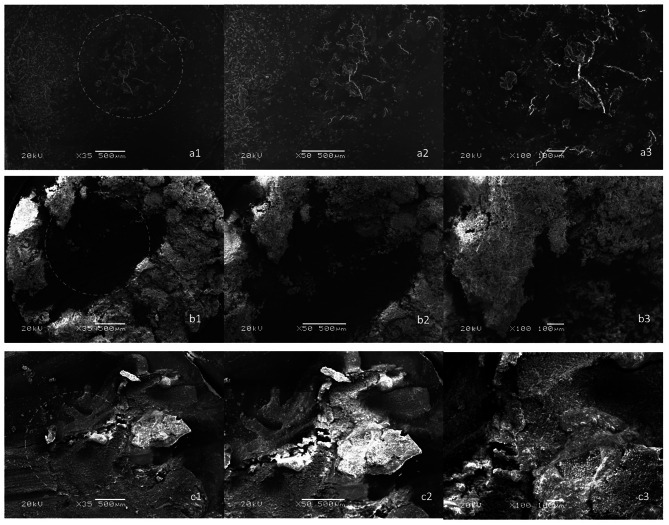
SEM images of failure types for Biodentine material. The fracture zone is marked with a circle. Images indicated with the letter ‘a’ represent adhesive fracture. a1 indicates x35 magnification, a2 indicates x50 magnification, a3 indicates x100 magnification. Images indicated with the letter ‘b’ represent cohesive fracture. b1 indicates x35 magnification, b2 indicates x50 magnification, b3 indicates x100 magnification. Images indicated with the letter ‘c’ represent mix fracture. c1 indicates x35 magnification, c2 indicates x50 magnification, c3 indicates x100 magnification

Failure types for specimens are as shown in Table 4. SEM images representing the failure types are shown in Fig. 4 for RetroMTA and Fig. 5 for Biodentine.

**Table 4:** Fracture Types in Different Groups

FSCSC	Time	Restorative Material	Fracture Types			
			Adhesive	Cohesive within FSCSC	Cohesive within restorative material	Mixed Adhesive Cohesive within FSCSC
RetroMTA	3 min	Composite Resin	6	2	0	2
		Theracal LC	10	0	0	0
		EQUIA Forte HT	10	0	0	0
	12 min	Composite Resin	5	3	0	2
		Theracal LC	10	0	0	0
		EQUIA Forte HT	10	0	0	0
	24 h	Composite Resin	6	1	0	3
		Theracal LC	10	0	0	0
		EQUIA Forte HT	10	0	0	0
Biodentine	3 min	Composite Resin	5	2	0	3
		Theracal LC	10	0	0	0
		EQUIA Forte HT	10	0	0	0
	12 min	Composite Resin	4	4	0	2
		Theracal LC	10	0	0	0
		EQUIA Forte HT	10	0	0	0
	24 h	Composite Resin	2	5	0	3
		Theracal LC	10	0	0	0
		EQUIA Forte HT	10	0	0	0

## Discussion

In the present study, three different restorative materials were placed after three different times over two different FSCSCs. Significant differences between the tested groups were confirmed. Therefore, the first null hypothesis was rejected. The results revealed that the RC restoration placed over Biodentine after 12 min, which is the setting time specified by the manufacturer, showed similar bonding efficiency compared to the one placed after 24 h. On the other hand, as the waiting time to place the RC over RetroMTA increased, the µSBS increased statistically. RC placed over any FSCSC used in the study had the highest µSBS compared to other overlaid restorative materials, regardless of time. Therefore, the second null hypothesis was also rejected.

Various types of adhesive materials have been preferred over FSCSC in previous studies. A previous study indicated that the shear bond strength (SBS) of Biodentine was lower when an one-step self-etch adhesive (SE) was used compared to a two-step total-etch (TE) system [20]. But, it has been suggested that acid etching of Biodentine might degrade the microstructure and could cause leakage through the biomaterial-composite interface [21]. A study evaluated the SBS of two-step TE, one-step SE and two step SE systems to Biodentine [22]. Although authors stated that the adhesive system did not affect the SBS statistically, they found that the highest SBS value was reached with two-step SE (Clearfil SE Bond). In another previous study, the SBS of a micro-hybrid RC placed resin over the Biodentine was evaluated using two-step TE, one-step SE containing 10-methacryloyldecyl dihydrogen phosphate (10-MDP) and one-step SE system not containing 10-MDP. The researchers stated that the SBS of the one-step SE system containing 10-MDP was superior compared to those of the other groups [23]. In a study evaluating µSBS of composite resin over Biodentine, no significant difference was found between using a universal adhesive in TE or SE strategies [24]. The authors also suggested that the functional monomer 10-MDP can bond to calcium of the calcium silicate cements in the same way it bonds to calcium of the tooth hydroxyapatite, thus enhancing chemical adhesion in addition to micromechanical adhesion. Based on this information, Clearfil SE Bond, a two-step SE adhesive system containing 10-MDP, was chosen because it is the gold standard adhesive for bonding to dentin [25]. 

There are two clinical approaches regarding the time for placement of RC over CSC, delayed (multiple-visit) and immediate (single-visit) placement [17]. In vital pulp treatments, completing the restoration in a single session reduces the risk of recontamination, avoids additional costs and time loss [18]. The purpose of delaying the placement of RC is to give the CSCs time to mature completely and acquire their ultimate physical and mechanical potential [17]. However, some studies have shown that CSC can absorb water from tissue moisture during maturation so the RC restorative procedure can be completed in a single visit [26, 27]. 

Although shorter compared to conventional CSCs, the initial setting time of Biodentine (12 min) is still considered a long period in clinical practice [9]. For this reason, in this study, 3-min waiting time was also examined, which is a more clinically acceptable time and representing a more immediate approach. This 3-min period is also the initial setting time of RetroMTA as stated by its manufacturer.

In a study examining the immediate (12 min) and delayed (7 days) bond strength results of Biodentine and ProRoot MTA (Dentsply Tulsa Dental Specialties, USA) with RC material, the bond strength of Biodentine at the two time intervals was not statistically different [18]. In another study, researchers performed restorations on Biodentine and Angelus MTA (Angelus, Brazil) after 3 min, 15 min and 2 days and examined their bond strength to RC; placing the RC on Biodentine at 3 different time intervals did not affect the bond strength [19]. The results of the mentioned study and the results of the present study are partially different. In the present study, the bond strength values of 3 min were found to be lower than those of the other placement times. This difference may be due to the fact that, unlike the present study, the SBS test was used in the mentioned study, not the µSBS test.

In a study in which RC restoration was performed over Biodentine using various adhesives; the researchers reported a significant difference between 12 min and 24 h bond strengths and stated that the 24 h values were higher [28]. The results of mentioned study are different from present study. In the present study, no difference was found between 12 min waiting time and 24 h waiting time for applying composite restoration over Biodentine. This difference may be due to the fact that the SBS test was used in the mentioned study, which is different from the present study, and 2-step SE was not preferred as an adhesive material.

In another study, the bond strength between Biodentine-RC was investigated using the µSBS test. Waiting times of 12 min, 24 h, and 1 wk were compared using different adhesive systems. The µSBS values were similar at all times in the groups using Clearfil SE Bond [29]. The results of the mentioned study support the present study. When the different adhesive groups of the mentioned study are analyzed, it is observed that the results vary according to the adhesive used [29]. This confirms that the difference in results between some previous studies and the present study is affected by the difference in adhesive system.

In a study similar to the present study, the SBS of RC restorations placed over Biodentine and RetroMTA was investigated. Biodentine was allowed to set for 12 min and RetroMTA for 10 min. The researchers then tested 4 different adhesive strategies. Clearfil SE Bond representing a two-step SE system, AQ bond Plus (Sun Medical, Japan) representing a one-step SE system, and Allbond Universal (Bisco Inc., USA) representing a universal adhesive system in which both total-etch and SE modes were tested. The specimens were subjected to SBS testing 24 h after placement of the composite restorations. It was reported that the Biodentine-RC bond strength was significantly higher than the RetroMTA-RC bond strength regardless of the adhesive strategies [30]. That result supports the findings of present study.

In the literature and in clinical practice, there is a reluctance to perform direct composite restorations on CSCs due to doubts about the setting time of CSCs. In previous studies, products from the glass ionomer family were highly preferred on CSCs before composite restoration for this purpose [9, 12, 31]. In addition, various materials such resin modified glass ionomer [19, 32], flowable RC [19], calcium hydroxide [33] have also been used investigated. In line with this information, the glass hybrid restorative material known as EQUIA Forte HT, one of the most advanced materials of the glass ionomer cement family, produced in 2019, was preferred in the present study. It has anticariogenic effects, is capable of remineralizing hard tissues, is self-adhesive, and has bulk-filling properties [34]. This material, like other GICs, can be applied on CSC before composite restoration. In addition, it allows for a permanent restoration, unlike traditional type GICs. Present study is the first in the literature to use EQUIA Forte HT over Biodentine and RetroMTA. The results of the present study showed that EQUIA Forte HT, when applied on both FSCSCs, showed lower bond strength compared to RC regardless of time. In a previous study, Biodentine was allowed to set for 12 min and then 2 RCs and 3 materials from the GIC family were applied [12]. The bond strength of the GIC family of restorative materials was found to be significantly lower than that of the RCs. One of the GIC family materials used in the aforementioned study is EQUIA Forte Fill (GC, Japan), which is an earlier version of glass hybrid restorative materials on the market [12]. These results support the present study, although the materials are different. In addition, it has been noted that GIC may absorb water when placed over freshly mixed CSC and may result in incomplete hydration and porosity of the CSC [33]. 

Theracal LC is another material considered as an intermediate material before composite restoration over FSCSCs in the present study. In fact, Theracal LC is also a CSC, contains polymerizable methacrylate monomers, is light-cured and allows the restoration to be completed in a single session in the VPT procedure [14]. It is indicated as direct and indirect pulp capping agent, base/liner material under class I-II restorations (under composites, amalgams, glass ionomer cements) [35]. Many studies have reported that the bond strength values of Theracal LC with resin composite materials are higher than those of other CSCs [3, 12, 36]. Therefore, the authors of the present study were curious about the effects of using FSCSCs and Theracal LC, which are essentially the same type of materials, together in order to protect the pulp in the best way and to obtain a strong bond strength to the composite restoration made in a single session, but they could not find a study on this subject in the literature. The present study showed that, in terms of bond strength, the application of Theracal LC on CSCs prior to RC has no advantage over the direct use of RC. Present study demonstrated that, in terms of bond strength, applying Theracal LC over CSCs prior to RC had no advantage over using directly RC.

Acceptable bond strength values varies in the literature. In some studies in the past, this value was accepted as 9 MPa [37], while in others it was accepted as 10–13 MPa [38, 39]. Some authors have stated that in order to be able to talk about an effective adhesion, it should be able to resist the contraction forces and for this purpose, it has been stated that a bond strength in the range of 17–20 MPa may be needed [40]. The bond strength values of present study were found to be lower than the stated values regardless of time. An acceptable threshold value for the bond strength of CSCs with restorative materials has not been reported in the literature. However, the results of the present study are consistent with previous studies [6, 30]. Bond strength between tooth tissues and restorative material is critical for a hermetic seal when performing the permanent restoration over CSCs. Since bonding capacity of CSCs to tooth is significantly lower than the bond strength between RC and tooth, CSCs should cover as limited an area as possible when placed as a VPT material to maximize the surface area for the RC.

Various methods are used to evaluate bond strength in dentistry, such as SBS, µSBS, tensile bond strength (TBS), and micro-tensile bond strength (µTBS) tests. µTBS and µSBS tests allow selection of specific tooth regions compared to conventional TBS and SBS tests. Bond strengths of less than 5 MPa are difficult to measure in the µTBS method and specimens can be easily damaged [41]. For this reason, the µSBS test method was preferred in this study.

It has been reported that adhesive failures are observed more frequently in micro bond strength tests than in macro tests because the force can be applied to a more specific area [42]. In the present study, adhesive failures were observed in all groups treated with Theracal LC and EQUIA Forte HT. For the RC groups, cohesive failures observed in FSCSCs could indicate that the bond strength between the materials were higher than the inherent strength of the FSCSCs.

Although attempts have been made to mimic oral conditions, the current study is an in vitro study. Pulp pressure and dentinal fluid from the dentinal tubules were not simulated. Therefore, in the current study, how FSCSCs behave when interacting with moisture from dentin was ignored. Therefore, clinical studies on these materials are needed. The conclusions reached within the limitations of this study can be summarized as follows:

1. For Biodentine material; there is no need to delay the restoration (24 h) in terms of bond strenght. However, it is necessary to wait 12 min, which is the setting time specified by the manufacturer, before performing a RC restoration.
2. For RetroMTA material; As the waiting time increased, the bond strength with the RC restoration increased.
3. Regardless of whether immediate or delayed approach is preferred, it is recommended to prefer direct RC restoration over FSCSC instead of using glass hybrid restorative material or Theracal LC (as intermediate material).

## Data Availability

The datasets used and/or analysed during the current study are available from the corresponding author on reasonable request.
